# *EGFR*突变调控肺腺癌PD-L1表达的分子机制研究进展

**DOI:** 10.3779/j.issn.1009-3419.2018.11.10

**Published:** 2018-11-20

**Affiliations:** 1 610041 成都，四川大学华西医院胸外科 Department of Thoracic Surgery, West China Hospital, Sichuan University, Chengdu 610041, China; 2 610041 成都，四川省肿瘤医院胸外科中心 Division of Thoracic Surgery, Sichuan Cancer Hospital and Institute, Cancer Hospital Affiliated to School of Medicine, University of Electronic Science and Technology of China (UESTC), Chengdu 610041, China

**Keywords:** 表皮生长因子受体, 程序性死亡配体-1, 信号通路, 肺肿瘤, Epidermal growth factor receptor, Programmed death ligand 1, Signaling pathways, Lung neoplasms

## Abstract

表皮生长因子受体酪氨酸激酶抑制剂（epidermal growth factor receptor tyrosine kinase inhibitors, EGFR-TKIs）和作用于程序性死亡受体-1（programmed death receptor, PD-1）/程序性死亡配体-1（programmed death ligand 1, PD-L1）的免疫检查点抑制剂是非小细胞肺癌（non-small cell lung cancer, NSCLC）治疗中具有里程碑意义的药物。然而，*EGFR*突变的NSCLC患者中PD-L1表达调控机制尚不完全清楚，可能涉及多条信号通路。本文就此进行综述，为探索*EGFR*突变和PD-L1表达之间的关系，以及指导创新性的NSCLC免疫化学治疗的策略提供科学依据。

## Abstract (en)

The epidermal growth factor receptor tyrosine kinase inhibitors (EGFR-TKIs) and programmed death receptor 1 (PD-1)/programmed death ligand 1 (PD-L1) immune checkpoint inhibitors were landmarks in the treatment of non-small cell lung cancer (NSCLC). However, the regulation mechanisms of PD-L1 expression were not fully clear in NSCLC patients with *EGFR* mutations. Multiple signaling pathways may be involved in the tumorigenesis regulation. This paper summarized and reviewed the potential *EGFR* mutations impacting on PD-L1 expression with aims to the development of strategies on immunochemical therapy for NSCLC.

肺癌是全球发病率最高的恶性肿瘤，也是癌症相关死亡的首要原因，中国每年新诊断的肺癌患者总数占全球的三分之一^[1, 2]^，已成为全球最重要的公共健康问题之一。针对驱动基因表皮生长因子受体（epidermal growth factor receptor, *EGFR*）突变的非小细胞肺癌（non-small cell lung cancer, NSCLC）患者，EGFR酪氨酸激酶抑制剂（tyrosine kinase inhibitors, TKIs）已成为一线治疗方案^[3]^。但常在治疗后的9个月-12个月，患者不可避免地出现耐药。这也是导致*EGFR*突变肺腺癌治疗失败和预后不良的主要原因。肺腺癌EGFR-TKIs耐药的发生是一个极为复杂的多因素、多途径、多层次的过程，目前的研究尚未达成共识。研究表明，约60% *EGFR*突变肺腺癌发生T790M二次突变可能是EGFR-TKIs耐药的主要机制^[4]^。另外，*c-Met*扩增、*AXL*激活、*EMT*激活以及IGF-1R信号通路的上调等也与EGFR-TKIs耐药性的产生密切相关^[4]^。虽然基于分子靶向药物等多模式的治疗极大改善了NSCLC患者的临床预后，但长期生存时间仍偏低，且复发和转移率高^[5]^，促使人们开始关注免疫治疗带来的潜在获益。程序性死亡受体-1（programmed death receptor 1, PD-1）/程序性死亡配体-1（programmed death ligand 1, PD-L1）抗体为*EGFR*突变或EGFR-TKIs耐药肺腺癌患者带来了治疗的曙光，但是目前在临床上进行免疫干预的认识及选择还是比较有限。因此，更好地理解EGFR-TKIs治疗后调控免疫应答的分子事件成为一个紧迫的主题。为此，本文对不同信号通路参与调控*EGFR*突变肺腺癌PD-L1表达的相关研究进行综述。

## 1 PD-L1

### 1.1 PD-L1的结构及分布

PD-L1也称为B7-H1（B7 Homolog 1）或CD274（cluster of differentiation 274），是PD-1的共抑制性配体。PD-L1由*PDCDL1*基因编码，位于染色体9p24.1。PD-L1被认为是B7蛋白家族的第三个成员，与B7.1和B7.2蛋白有15%-20%的同源性，1999年由Dong等首次将其命名为B7-H1。PD-L1的全长包括7个外显子编码区，对应一个含有290个氨基酸40 kDa长度的蛋白质。PD-L1是由胞外区IgV样和IgC样结构域，一个疏水跨膜结构域，以及30个氨基酸构成的一个短胞质尾区组成的Ⅰ型跨膜蛋白，但其信号传导作用仍不是很清楚^[6, 7]^。PD-L1可表达于造血细胞，包括T细胞、B细胞、巨噬细胞、树突状细胞（dendritic cells, DCs）和肥大细胞中。也可表达于非造血的健康组织细胞中，包括血管内皮细胞、角化细胞、胰岛细胞、星形胶质细胞、胎盘滋养细胞、角膜上皮和角膜内皮细胞。同时，PD-L1还可在肿瘤细胞和肿瘤间质中表达。

### 1.2 PD-L1的生物学功能

PD-L1通过结合PD-1受体形成的PD-1/PD-L1通路在维持中枢和外周免疫耐受方面起到重要作用^[8]^。PD-L1在胸腺和树突状细胞中高表达，相互作用的PD-L1/PD-1会阻止幼稚T细胞的增殖和分化^[9]^，PD-L1还和CD80相互作用，从而对激活的T细胞产生负性调节作用^[10]^。其次，PD-1/PD-L1还参与免疫耗竭的生理机制，即在抗原持续存在的情况下诱导效应T细胞凋亡，抑制T细胞复制和成熟，以防止在慢性感染中出现组织破坏^[11]^。同时，PD-L1参与抗肿瘤免疫反应的调节，在肿瘤浸润淋巴细胞中普遍发现PD-1持续性上调，而恶性肿瘤细胞会利用PD-L1的表达来逃避免疫系统对其造成破坏^[12]^。

## 2 *EGFR*突变调控肺腺癌PD-L1表达的相关信号通路

现认为，约20%-30% NSCLC中PD-L1的肿瘤比例分数（tumor proportion score, TPS） > 50%，其表达导致肿瘤免疫微环境改变，促进肿瘤细胞更具侵袭性和免疫逃逸^[13]^。亚洲人群*EGFR*突变率（47%）较非亚洲人群（13%-15%）更常见，主要为19外显子缺失（del746_A750）或21外显子点突变（L858R）^[14, 15]^。目前，*EGFR*突变与PD-L1表达调控尚存在争议，但是体外实验及大中心研究均提示*EGFR*突变导致NSCLC中PD-L1表达异常升高。2014年，Azuma等^[13]^在*Annals of Oncology*发表研究发现，PD-L1高表达与*EGFR*突变关系密切且与影响NSCLC患者生存预后，且PD-L1 TPS≥50%与*EGFR*突变的相关性已在多项研究^[16-18]^中得以证实。但近期也有研究^[19]^显示PD-L1在EGFR野生型肺癌组织中高表达，其表达与*EGFR*突变并无显著相关性。从药物治疗造成癌细胞最初损伤到其最终死亡之间存在着多条信号传导通路组成的网络式调控体系^[20]^。因此，*EGFR*作为NSCLC重要致癌基因和药物靶点，更好地理解*EGFR*突变通过不同相关信号通路直接或间接调控PD-L1表达参与肿瘤免疫逃逸尤为重要。

### 2.1 PI3K/AKT/mTOR信号通路

NSCLC中EGFR可以激活PI3K/AKT/mTOR信号通路，它能增加细胞增殖、代谢和存活能力，并在肺癌形成过程中起到关键作用。*EGFR*突变的NSCLC细胞中，当PI3K、AKT和mTOR表达同时下调，PD-L1的表达随时间出现显著下降^[21, 22]^。在*EGFR*突变敏感细胞中使用EGFR-TKIs也会导致PD-L1表达下降。EGFR-TKIs不仅使EGFR磷酸化减弱，同时也降低AKT的磷酸化，从而进一步调节PD-L1的表达。下调PI3K表达后，AKT也出现磷酸化水平降低并最终导致PD-L1表达下降^[17, 21, 23]^。AKT/mTOR信号通路可在*EGFR*突变的肺腺癌中激活，且mTOR可独立调节PD-L1的表达^[22]^。此外，γ-干扰素（interferon-gamma, IFN-γ）不仅起到重要的抗肿瘤作用，同时也可激活PI3K-AKT和JAK2-STAT1通路，抑制PI3K后不仅可下调PD-L1表达，同时可增强IFN-γ抗肿瘤增殖的能力，说明PI3K-AKT和JAK2-STAT1通路间存在一定交叉效应^[24]^。说明了PI3K/AKT/mTOR信号通路在*EGFR*突变NSCLC中对PD-L1的表达起到调节作用。但也有研究^[25]^显示*EGFR*突变后AKT磷酸化激活，且抑制AKT磷酸化后并未出现PD-L1表达的变化，但其可能机制仍不明确。

### 2.2 MAPK信号通路

MAPK中的MEK/ERK通路通常由肿瘤异常上游信号酪氨酸激酶受体扩增或突变而激活。有证据表明MEK/ERK通过炎症信号通路交叉调节PD-L1的表达^[26]^。在*EGFR*突变NSCLC细胞中也发现MEK-ERK信号通路对PD-L1表达起到调控作用^[23, 27]^，激活ERK1/2通路可显著上调PD-L1的表达^[25]^。但在一些研究中发现*EGFR*突变细胞中抑制ERK并未出现MAPK信号通路对PD-L1表达的调控现象^[21]^。即使在多个肺癌细胞系，包括EGF诱导PD-L1过表达细胞株中，使用MEK抑制剂也未发现PD-L1表达发生变化^[17, 22]^。说明MAPK信号通路并不是直接调控*EGFR*突变NSCLC的直接通路。

### 2.3 JAK/STAT信号通路

JAK/STAT是肿瘤发生、发展和形成的一个经典信号通路。无论在*EGFR*突变TIKs敏感/耐药细胞株中，抑制JAK/STAT3后均出现PD-L1表达下降^[21]^。但使用EGFR-TKIs调节PD-L1表达时，只有使STAT3去磷酸化才会出现PD-L1表达下降，而直接沉默STAT3后并未发现PD-L1表达的变化^[23]^。在EGF诱导的EGFR和STAT3磷酸化和激活细胞中，分别抑制和沉默STAT3和JAK2，PD-L1无论在mRNA水平还是蛋白水平均出现了显著下降，提示JAK/STAT3通路在其中起到了重要作用^[17, 28]^。

### 2.4 NF-κB信号通路

NF-κB作为转录因子家族，可在肿瘤突变癌基因和炎症微环境产生的细胞因子下被激活。研究^[29]^发现NF-κB的亚基RELA（p65）可以在NSCLC细胞中结合PD-L1的启动子，并以RELA-MUC1-C复合体的形式直接调控PD-L1的转录。NF-κB作为EGFR激活的重要下游通路，调节肿瘤细胞的增殖和化疗耐药。在*EGFR*突变细胞系中表达高于EGFR野生型，且与PD-L1表达呈正相关。当在*EGFR*突变细胞系中沉默NF-κB后，发现随着NF-κB的亚基RELA（p65）的沉默，PD-L1的表达随之衰减，再次证明*EGFR*突变NSCLC中PD-L1表达的升高需要NF-κB的参与^[30]^。

### 2.5 其他调控机制

Yes相关蛋白1（Yes associated protein 1, YAP）是Hippo通路上的效应因子，是许多癌症中的致癌基因。作为共转录因子会和TEAD形成一个复合物，其具备DNA结合域并调节多个基因在细胞增殖、凋亡抑制、上皮间质转化和其他方面的功能^[31]^。虽然YAP可能不会调节PD-L1的上游通路，但研究表明YAP可直接结合在PD-L1启动子上从而调节其表达。在*EGFR*突变细胞株过表达YAP可增加PD-L1的表达，敲减YAP后PD-L1表达下降，而过表达PD-L1则对YAP的表达没有影响。通过染色质免疫共沉淀技术（ChIP）发现YAP/TEAD结合在PD-L1启动子区域，因此YAP是通过增加PD-L1的转录来调节其表达^[32]^。同时，YAP表达参与乏氧环境中的肿瘤进展^[33]^，在*EGFR*突变NSCLC中通过凋亡通路影响PD-L1调节EGFR-TKI的耐药^[34]^。

桥接整合因子-1（bridging integrator-1, BIN1）是一种MYC适配体蛋白，具有抑癌特性^[35]^。BIN1在NSCLC的*EGFR*突变细胞株中表达降低或缺失，而PD-L1在BIN1过表达后明显下降，敲低BIN1后PD-L1表达明显上调。无论体外还是体内实验均证实BIN1的过表达可使EGFR/MAPK信号通路失活从而抑制和调控PD-L1介导的免疫逃逸^[36]^。

## 3 PD-1/PD-L1抑制剂在*EGFR*突变NSCLC中的应用

如前所述，*EGFR*突变可能通过不同信号通路影响PD-L1表达。*EGFR*突变NSCLC患者中EGFR-TKI获得性耐药也会导致PD-L1表达的明显升高^[37]^，在经过EGFR-TKI治疗的患者中，获得性MET阳性表达也参与了PD-L1表达升高的调控，在肺癌细胞中可能是通过miR-200/ZEB1进行调控^[38]^。那么PD-L1是否可以作为一个重要的*EGFR*突变NSCLC中免疫原性评价指标，继而指导这类患者可能从免疫检查点抑制剂或联合EGFR-TKI治疗中获益。过往针对免疫检查点抑制剂的临床研究纳入标准仅考虑PD-L1阳性一方面，随着对EGFR突变调控PD-L1表达相关分子机制的不断认识，针对*EGFR*突变患者抗PD-L1药物疗效的临床数据也在不断增多。Keynote-001作为Ⅰ期临床试验发现*EGFR*突变NSCLC中使用pembrolizumab治疗PD-L1阳性（TPS≥50%）和（TPS 1%-49%）患者的客观缓解率（overall response rate, ORR）分别为20%和8.7%，而在PD-L1阴性（TPS < 1%）的情况下，ORR为0%^[39]^。在单臂、开放标签Ⅱ期涉及亚洲、欧洲和北美多中心临床研究ATLANTIC中，应用durvalumab治疗*EGFR*突变阳性、PD-L1高表达（TPS > 25%）的晚期或治疗进展的NSCLC患者中，ORR为14.1%，明显高于PD-L1低表达（TPS < 25%）的3.6%^[40]^。由于缺乏针对pembrolizumab在*EGFR*突变和非突变NSCLC患者中的临床疗效证据，一项Ⅱ期临床试验（NCT02879994）对*EGFR*突变阳性且没有接受过EGFR-TKI、晚期NSCLC及PD-L1阳性（> 1%，22C3抗体）的患者给予pembrolizumab治疗。非常遗憾，由于缺乏治疗有效性，该试验入组11例患者后即终止。结果表明pembrolizumab在未接受TKI治疗、PD-L1阳性（包括TPS > 50%）的*EGFR*突变阳性晚期NSCLC患者中并不适用^[41]^。

在联合用药方面，Checkmate-012研究评估了nivolumab联合化疗/erlotinib/ipilimumab或作为单药治疗NSCLC患者的安全性和耐受性。作为该研究的一部分，在nivolumab联合erlotinib治疗组中，入组21例患者经过联合用药直到出现疾病进展或无法耐受的毒性反应，其ORR为19%，24周的无进展生存（progressive free survival, PFS）率为51%，18个月OS率为64%。其中20例EGFR-TKI经治患者中3例达到部分缓解（partial response, PR），且中位缓解时间为60.1个月，1例EGFR-TKI初治的患者达到PR并在文献报道时已持续接受了72.3个月的治疗^[42]^。在多臂、开放标签的Ⅰb期TATTON试验中，对Osimertinib联合durvalumab治疗EGFR-TKI敏感突变患者的效果进行评估。其中Osimertinib是一个强效、不可逆的EGFR-TKI药物，主要作用于EGFR-TKI敏感突变和T790M突变引起的继发耐药。在23例EGFR-TKI经治患者中，T790M突变阳性的ORR为67%，EGFR初治患者ORR达到70%，但由于治疗期间肺间质疾病（interstitial lung disease, ILD）的高发生率（13/34, 38%），后续试验入组已经暂停^[43]^。另外，还有很多联合治疗的相关临床试验还在入组中，如NCT02039674、NCT02088112、NCT1454102、NCT02574078等。

## 4 展望

综上所述，改善中晚期NSCLC患者生存问题一直是胸部肿瘤领域亟待解决的难题。*EGFR*突变可激活NSCLC细胞中的不同信号通路，介导PD-L1表达升高，从而导致免疫逃逸和治疗失败。但关于*EGFR*突变与PD-L1表达的相关研究仍处于起步阶段，诸多未知因素尚有待解决。相信随着对相关研究的不断深入，将为PD-1/PD-L1抑制剂在*EGFR*突变NSCLC患者中单药或联合EGFR-TKIs的多模态免疫-靶向治疗策略中提供理论基础，以期改善未来NSCLC的治疗预后。
